# Preliminary In Vivo Ocular Tolerance Assessment of a Cefuroxime Sodium Suspension in Self-Emulsifying Oil

**DOI:** 10.3390/pharmaceutics17101320

**Published:** 2025-10-11

**Authors:** Katarzyna Krzemińska, Eliza Wolska, Juliusz Chorążewicz, Małgorzata Sznitowska

**Affiliations:** 1 Department of Pharmaceutical Technology, Medical University of Gdansk, Hallera 107, 80-416 Gdansk, Poland; katarzyna.krzeminska@gumed.edu.pl (K.K.);; 2 Department of Ophthalmology, Medical University of Gdansk, Smoluchowskiego 17, 80-214 Gdansk, Poland

**Keywords:** self-emulsifying oil, antibiotics, cefuroxime sodium, eye drops, tolerance

## Abstract

Cefuroxime sodium (CEF) is a second-generation cephalosporin that remains unstable in an aqueous environment. The answer to this low stability may be self-emulsifying oils, which are isotropic mixtures of oil and surfactants, in which the stability of CEF has already been proven. Self-emulsifying oils are well known for their ability to enhance the solubility and bioavailability of lipophilic drugs. This research presents a preliminary in vivo study of an innovative approach to develop eye drops in the form of a self-emulsifying oil (SEO) containing suspended water-labile antibiotic cefuroxime sodium. Such a concept has never been explored before. Upon contact with tear fluid, the preparation rapidly forms an emulsion, allowing for the rapid dissolution of the antibiotic. The aim of the study was to assess the tolerability of such eye drops. CEF (5% *w*/*w*) was suspended in SEO carriers, prepared by dissolving surfactants (Tween 20; 5% *w*/*w*) in Miglyol. The in vivo evaluation was conducted on rabbits after two once-a-day applications of the eye drops. The study demonstrated the safety of both the SEO-placebo and the SEO containing suspended CEF. The formulations did not affect the appearance of the cornea and iris. During the observations, only changes in the conjunctiva of the eye were noted, which manifested as conjunctival hyperemia. The result of the Draize test was an average of 3.3 points out of 110 possible points, which classifies the CEF-SEO suspension as minimally irritating.

## 1. Introduction

The internationally recognized standard test for acute eye toxicity is the Draize test on rabbits, which was developed in the 1940s by the Food and Drug Administration (FDA) [1]. It has also been accepted by the Organization for Economic Cooperation and Development (OECD).

Scientific studies have shown that the rabbit is commonly regarded as a sensitive model for humans, which can be considered a positive aspect, as it adds a safety margin when assessing the risk of an irritating effect in humans. Compared to humans, rabbit corneas are thinner, have lower tear production, a higher blink rate, and greater ocular surface sensitivity. Rabbits also have larger conjunctival sacs and a nictitating membrane (third eyelid), which may facilitate the removal of the tested substance from the eye surface [2]. Due to the high sensitivity of the rabbit eye, it is possible to observe a false-positive irritation effect that would not occur in humans, leading to a substance being classified as irritating even though such an effect would not be observed in humans. In the absence of an irritation effect, there is confidence that a preparation is safe and can be used in humans. White New Zealand rabbits are most commonly used in tests because they have large eyes with well-described anatomy and physiology, are easy to tame, and are readily available [1].

The Draize test procedure involves applying 0.1 mL of the test material to the eye of a conscious rabbit for up to 72 h, with the other eye serving as an untreated control [1,3,4]. The original Draize test protocol required the participation of at least six rabbits. Now, this number has been reduced to three animals or even one animal when severe eye damage is expected. Pathological changes are assessed in the cornea, conjunctiva, and iris (ciliary body), following the same evaluation criteria as in the Draize test.

Although there are known alternatives to the Draize test, such as ex vivo organotypic eye models, in silico prediction models, and 3D reconstructed human cornea-like epithelium (RhCE), animal studies remain essential for confirming the safety of a preparation before it can be approved for human use. Restrictions on animal use primarily apply to toxicological studies, as no government institution has yet eliminated the use of animals in fundamental biomedical research or drug development [3].

The purpose of this study was to evaluate the tolerance towards eye drops formulated as a self-emulsifying oil (SEO) composed of Miglyol and surfactant: Tween 20 (5% *w*/*w*), containing suspended and micronized cefuroxime sodium (CEF) in a 5% (*w*/*w*) concentration and citrate sodium (2% *w*/*w*). In the tested formulation, the upper limit of the concentrations of both CEF and excipients commonly used in ophthalmic preparations was applied to demonstrate the potential maximum irritant effect that might occur following ocular administration of this dosage form. Sodium citrate was added as a deflocculant, facilitating redispersion of the sediment after short shaking.

Such a novel formulation could serve as an alternative to extemporaneously compounded aqueous eye drops. Eye drops with CEF are used in ophthalmology to prevent and treat bacterial infections of the eye, particularly those caused by *Staphylococcus* spp. and *Streptococcus* spp. Although CEF is most commonly administered as an intracameral injection during cataract surgery to reduce the risk of postoperative endophthalmitis, it may also be compounded into 5% ophthalmic drops (50 mg/mL). Such high-concentration preparations are prescribed in selected clinical situations, including *Neisseria* conjunctivitis, ophthalmia neonatorum, bacterial keratitis, blebitis, bleb-related endophthalmitis, ocular trauma, or postoperative infections, where strong local antibacterial activity is required [5]. However, CEF is unstable in water, and these eye drops must be extemporaneously prepared, with a shelf life limited to 24 h at room temperature or up to 14 days under refrigeration, which considerably restricts their clinical applicability [6]. Similar to other water-labile antibiotics, such as cephalosporins, glycopeptides, penicillins, and polypeptides, pharmaceutical compounding remains the only available method of preparation of these eye drops for clinical use.

The solution to the problem of CEF instability may be the use of self-emulsifying oil (SEO) as a vehicle, which is a water-free system consisting of oil and a surfactant [7]. These carriers are primarily studied for their potential to improve the solubility and bioavailability of active pharmaceutical ingredients [8], whereas our research aims to develop SEO eye drops in the form of a suspension containing water-unstable antibiotics to enhance their stability [9]. As shown in previous studies, CEF remains stable in the developed SEO carrier for up to two years at room temperature (60% relative humidity) and one year at 40 °C (75% relative humidity), with stability defined as a loss of no more than 10% of initial content. No peaks indicating the presence of degradation products were observed in the HPLC chromatograms, while the analysis demonstrated complete degradation of the antibiotic in aqueous solution within seven days at room temperature [9].

An alternative carrier for water-sensitive active substances could be an oil, but oily eye drops are disturbing to vision and are not well accepted by patients. In contrast to oily solutions, upon contact of SEO with tears, an emulsion is rapidly formed, which is better tolerated and does not disturb vision. Good tolerance of SEO has already been demonstrated in animals [10], and the use of commercial eye drops in the form of emulsion also supports the concept of safety for such in situ-formed emulsion. Products in the form of oil-in-water (o/w) emulsions have already been introduced to the market for treating dry-eye syndrome (Ikervis^®^), glaucoma (Xelpros^®^), or to achieve a hydrating and lubricating effect (Cationorm^®^).

The microbiological efficacy of CEF in SEO has been confirmed for freshly prepared formulations and formulations stored at room temperature for one year [9]. Emulsions prepared from the given formulations, diluted 3:1 with water, were applied to plates with agar containing *Staphylococcus aureus*, along with a 5% CEF aqueous solution as a reference. No statistical differences were observed between the inhibition zone sizes of the resulting emulsion and the tested 5% CEF standard solution, which formed the basis for assessing irritation in the in vivo model.

**Figure 1:**
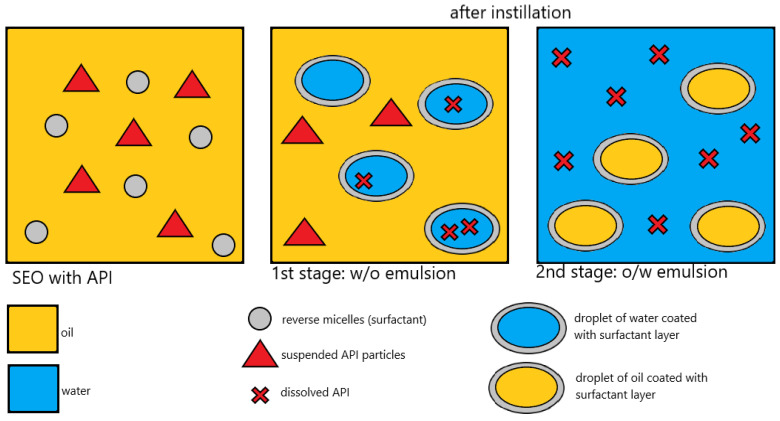
Schematic depiction of the transformation of the SEO containing suspended CEF into an emulsion after contact with water or tear fluid [11].

SEO containing suspended antibiotics has not yet been studied in vivo, which thus became the objective of the conducted experiment. The antibiotic, used as a hydrophilic salt, is suspended in SEO but dissolves fast when in contact with tears, which means that, after administration to the eye, a formulation that is suspended in an oily vehicle is immediately transformed into a solution of antibiotic in o/w emulsion (Figure 1) [11]. Due to the presence of oil, a prolonged in vivo residence time and effect may be an additional advantage of SEO.

Previous studies have demonstrated good tolerance of SEO-placebo systems [12]. Furthermore, a comprehensive ocular irritation test was performed in which SEO eye drops containing dissolved cyclosporine A as the drug substance were administered to rabbits, confirming the absence of any irritant effects [10]. In the current study, the effect of the suspension-type SEO eye drops is investigated.

## 2. Materials and Methods

### 2.1. Materials

Cefuroxime sodium (CEF) powder for injection was purchased from MiP-Pharma (Chephasaar, St. Ingbert, Germany). Polyoxyethylene sorbitan monolaurate (Tween 20) was purchased from Sigma-Aldrich (Steinheim, Germany), and fractionated pharmacopoeial coconut oil (Miglyol 812—MCT, Medium Chain Triglycerides) was purchased from Caesar & Loretz (Hilden, Germany). Sodium citrate (2% *w*/*w*) was obtained from Stanlab (Lublin, Poland).

### 2.2. Tested Preparations

The suspensions were prepared from sterile ingredients under aseptic conditions in a vertical airflow cabinet with a HEPA filter (model CR-VCB001, Recco, Wloclawek, Poland).

The SEO-placebo carriers were made by mixing Miglyol with Tween 20 at a 5% (*w*/*w*) concentration, using a magnetic stirrer at 50 °C, and subsequently sterilized by filtration through a Stericup^®^ Quick Release system (Millipore, St. Louis and Burlington, MA, USA) equipped with a 0.22 µm polyethersulfone (PES) membrane.

**Figure 2:**
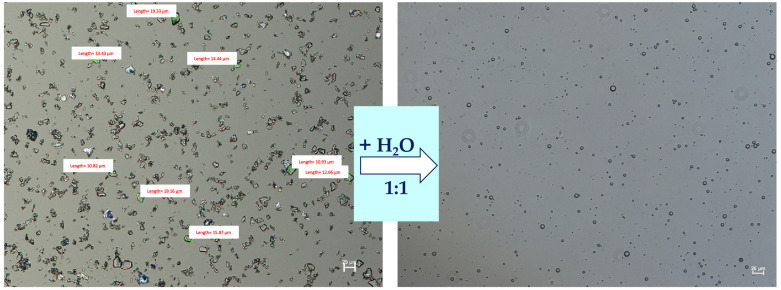
Microscopic image of CEF suspended in SEO and emulsion obtained after contact with water (1:1 *w*/*w*) (scale bar 25 µm).

Sterile cefuroxime sodium and sodium citrate were micronized in a jet mill (LaboMill, Food Pharma Systems, Como, Italy). Then, the CEF-SEO formulations were prepared by mixing SEO-placebo, CEF (5% *w*/*w*), and sodium citrate (2% *w*/*w*) in a Planetary Mixer aRe-250 (Thinky, Tokyo, Japan) for two cycles, each lasting 2 min at 2000 rpm. In the eye drop CEF-SEO formulation, the suspended CEF particles were mostly below 10 µm in size, with no particles exceeding 25 µm. Upon contact with an aqueous environment at a 1:1 (*w*/*w*) ratio, the formulation formed emulsion droplets, as presented in Figure 2.

### 2.3. Eye Test

With the approval of the ethics committee, following the ARVO Statement for the Use of Animals in Ophthalmic and Vision Research, the in vivo toxicological studies were conducted using the low-volume eye test (LVET) method, which represents a refinement of the Draize test [4]. According to the LVET procedure, the volume applied is 10 µL, but this was increased in our study to 25 µL, which corresponds to the volume of a single drop administered to the eye in clinical practice.

The experiment was performed on New Zealand’s white rabbits (males) weighing 3.5–4.5 kg. One drop (25 µL) of SEO containing 1.25 mg of CEF (SEO-CEF; 5% *w*/*w*) was administered directly onto the corneal surface of the rabbits. Reference preparations were as follows: 0.9% NaCl solution, SEO-placebo (no active substance), or 5% *w*/*w* aqueous solution of CEF. Each of the four preparations was administered twice to 6 eyeballs with an interval of 24 h. The drop administration scheme is shown in Table 1.

An ophthalmic examination was conducted before and after 30 min, 1 h following each application. The degree of irritation (redness, pathological discharge) and changes in the eye tissues (clouding, swelling, and epithelial defects) were assessed visually and documented photographically. The assessment was performed according to the Draize eye irritation test scoring scale, while the eye irritation was evaluated based on changes occurring in the cornea, iris, and conjunctiva [1]. The irritant effect of the tested compounds was determined based on the number of points obtained according to the classification by Kay and Calandra (Table 2). The final score is obtained by adding up the points, and the maximum possible score is 110.

## 3. Results

**Figure 3:**
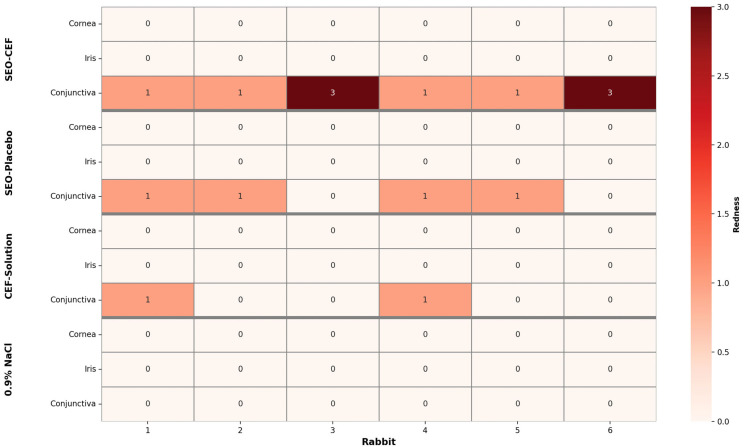
Observations of the eyes made 30 min after the instillation of eye drops (second day of the conducted study).

Irritation assessments were performed six times over two days: on the first day, the assessments were made before administering the drops and then 30 min and 1 h after application. On the second day, evaluations occurred before the drops (24 h after the previous application) and, similar to the first day, 30 min and 1 h post-application. Figure 3 presents, as an example, the results obtained during the observation of eyes 30 min after the application of eye drops on the second day.

The results obtained on the first and second days were the same. What should be noted is that after 1 h, the irritation was assessed at exactly the same levels as after 30 min, and after 24 h—before the next scheduled observation—no redness of the eyeball was observed in any animal.

Table 3 demonstrates summarized scores of observations made during the study.

**Figure 4:**
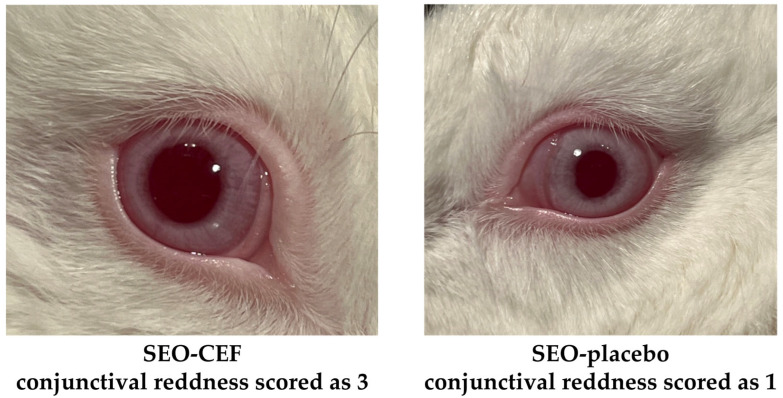
Representative images of rabbit eyes showing conjunctival redness on the second day of the study, 30 min after instillation of SEO containing CEF (**left**) and SEO-placebo (**right**).

As a result, none of the tested preparations affected the appearance of the cornea and iris in any of the rabbits. During the observations, only changes in the conjunctiva were noted, manifested as hyperemia or redness. The observed changes were more pronounced in the case of SEO formulations (placebo and CEF suspension) than in the aqueous CEF solution. A total of 0.9% NaCl was used as a reference, and as expected, did not induce irritation in any of the animals. A representative example of the observed reactions, illustrating the intensity of hyperemia, is presented in Figure 4.

Application of both the SEO-placebo and CEF-SEO led to increased blinking or partial eye closure, which was observed for approximately 20 s, most likely due to the need for emulsification of the formulation. Following the administration of the aqueous CEF solution, ocular redness was observed in two eyeballs, which were rated 1, whereas after the administration of the SEO-placebo, it was noted in four eyeballs, also rated 1. In all instances, the redness was classified as mild, corresponding to a score of one out of three possible levels. SEO-CEF affected all six eyeballs in which the formulation was applied. These described changes were also visible during the follow-up examination 1 h after application. However, after 24 h, redness was no longer observed in any tested animals.

Both at 30 min and 1 h after application, the eyeballs were clear, with no solid particles on the ocular surface and with no signs indicating incomplete antibiotic dissolution. No accumulation of residues from the in situ-formed emulsion was observed in the corner of the eye, which can occasionally occur following the administration of eye drops in such a form.

Considering both visual observations and behavioral assessments of the rabbits during and immediately after the administration of the tested formulations, it can be inferred that they did not cause any significant discomfort to the rabbits’ eyes. As demonstrated in Table 3, the result of the Draize test was an average of 3.3 points out of 110 possible points, which classifies the SEO suspension as minimally irritating to the rabbits’ eyes [4]. The SEO-placebo formulation was found to be less irritating: four eyes showed mild irritation, and were scored as 1, resulting in a mean score of 1.33 ± 1.03, which classified this formulation as practically non-irritating. Similarly, the CEF solution had a mean score of 0.66 ± 1.03.

## 4. Discussion

The conducted study is the first attempt to apply the SEO carrier in vivo with the aim of enhancing the stability of water-labile antibiotics. The results represent only preliminary findings from an ocular irritation study following the administration of the SEO with the suspended active substance to the eye, whereas, to date, only studies on solution-type SEO formulations have been conducted, aiming to improve the solubility and bioavailability of lipophilic drugs. The concept described in this work involves an active substance that is highly soluble but unstable in an aqueous environment, which is an innovation. Long-term stability of CEF in SEO has been proven based on the chromatographic analysis and antimicrobial efficacy tests.

An undoubted advantage of the proposed formulations is the fact that the suspension form can be produced on an industrial scale without limitations related to stability or drug concentration, which typically occur with aqueous solutions. Importantly, all the ingredients used have been recognized as safe and are already in use in ophthalmic preparations.

A technological novelty of this work also lies in the use of sodium citrate, which is suspended in SEO and acts as a deflocculating agent. Such a function of these excipients is well proven in aqueous suspensions [13], but we also demonstrated its activity in the oily dispersing phase, even if the compound does not dissolve [9].

Because the current guidelines encourage replacing animal testing with alternative methods such as cell-based assays or ex vivo chicken eye models, this study was limited to a small group of animals—only six eyes—in keeping with the 3R principle (replacement, reduction, and refinement). Although the use of animals is discouraged whenever possible, regulatory requirements for the registration of a new medicinal product still require certain in vivo safety evaluations, making a limited animal study unavoidable in this case [2,4]. Ocular preparations, especially, require an animal tolerance test before the first use in humans. For this reason, published studies evaluating ocular irritation in rabbits are typically conducted, although they use a limited number of animals [14,15].

The results obtained in the current study prove that the suspension of CEF in SEO is safe, as the scores from the Draize test were low. The SEO suspension obtained an average score of 3.33 ± 2.06, which is higher than observed after the SEO-placebo or CEF aqueous eye drops were used in clinical practice, but it still classifies the novel formulation as only minimally irritating. Moreover, the reaction was limited to reversible hyperemia, while the cornea and iris were unaffected. It is worth noting that rabbits are a highly sensitive model, often reacting more than the human eye. This means that the irritation observed in rabbits may be significantly less severe in humans.

It should also be noted that the assessment of conjunctival redness is somewhat subjective and may vary between researchers. Particularly stringent criteria were adopted in the tests conducted: one point was awarded even for slight redness of the eyeball, visible after administration, and three points meant clear redness of the entire eyeball. By contrast, the literature analysis shows that in many other studies, a score of one is often given in cases of much more pronounced redness, which in our system would correspond to a three [16]. Our restrictive classification method was deliberately adopted in order to maximize the safety of future use of the product by ensuring the most conservative assessment of potential irritation. However, as can be seen in Figure 4, the difference in redness of the eyeball, despite the extreme ratings of one and three, is practically imperceptible in the photo.

The enhanced conjunctival redness in response to the SEO-CEF formulation may be attributed to the solid particles present in the SEO-CEF suspension, although their size was in the range below 25 µm, which is generally accepted in ophthalmic preparations [17]. It is also possible that the observed hyperemia may have been caused by the rapid dissolution of the suspended sodium citrate and antibiotic particles, occurring immediately after contact of SEO-CEF with tear fluid, which may be accompanied by thermal effects.

The proposed SEO formulation represents a novel approach to self-emulsifying ocular systems, as it is designed as a suspension of a highly water-soluble drug that is unstable in aqueous environments—a concept not previously described in the literature. Owing to the presence of a lipophilic phase, the SEO suspension is expected to remain on the ocular surface for a longer period than a conventional aqueous solution, potentially enhancing drug residence time and bioavailability. Compared with emulsions, a key advantage of this system lies in its ability to provide long-term stability of the active substance, which would rapidly degrade in water and, therefore, could not be formulated as a stable solution or emulsion. This innovative suspension thus combines the benefits of both suspensions and emulsions: like a suspension, it allows the incorporation of drugs without solubility constraints and ensures chemical stability, while upon contact with tear fluid it immediately dissolves and self-emulsifies, promoting ocular tolerance similar to that of an emulsion and minimizing irritation typically associated with conventional suspensions. Despite its simple manufacturing process, the SEO suspension successfully integrates the strengths of related ocular dosage forms while eliminating many of their inherent drawbacks.

We would like to emphasize that the present study was intended as a preliminary step toward in vivo investigations of the ocular tolerance of SEO formulations containing antibiotics. These initial experiments were designed to provide a first confirmation of safety and, in the event of good tolerability, to serve as the basis for comprehensive irritation testing and in vivo pharmacokinetic studies that are essential for the new drug approval process.

## 5. Conclusions

While further in vivo studies are necessary, the results of the conducted analyses strongly suggest that SEO formulations with the water-labile antibiotic suspension represent a promising therapeutic approach and are likely to be effectively applied in humans, which is expected to be confirmed in subsequent stages of in vivo research on a larger group of animals.

## 6. Patents

European Patent No. EP3917498: Self-emulsifying pharmaceutical composition in the liquid form, containing as the active substance a drug substance unstable in an aqueous environment. Sznitowska M., Bączek T., Sych A., Krzemińska K. European Patent Office 2025 [11].

## Figures and Tables

**Table 1:** Administration schedule of eye drops in rabbits.

Left Eye	Rabbit	Right Eye
SEO-CEF	1	CEF-Solution
0.9% NaCl	2	SEO-CEF
SEO-CEF	3	SEO-placebo
CEF-Solution	4	0.9% NaCl
SEO-placebo	5	CEF-Solution
0.9% NaCl	6	SEO-placebo
SEO-CEF	7	CEF-Solution
0.9% NaCl	8	SEO-CEF
SEO-CEF	9	SEO-placebo
CEF-Solution	10	0.9% NaCl
SEO-placebo	11	CEF-Solution
0.9% NaCl	12	SEO-placebo

**Table 2:** The Draize scoring system for assessing the severity of eye injuries [4].

Eye Tissue	Scale	Maximum
Cornea	Opacity (1–4) × Area (1–4) × 5	80
Iris	Grading value (1–2) × 5	10
Conjunctiva	[Redness (1–3) + Chemosis (1–4) + Discharge (1–3)] × 2	20
Total score		110

**Table 3:** Scores noted in 6 eyeballs of rabbits 30 min and 1 h after administration of the tested formulations (0—no changes). Data are presented as mean ± SD (n = 6).

	SEO-CEF	SEO-Placebo	Solution-CEF	0.9% NaCl
Cornea				
-opacity	0	0	0	0
Iris	0	0	0	0
Conjunctiva				
-redness	one in four eyes	one in four eyes	one in two eyes	0
(max. 3)	three in two eyes			
-chemosis	0	0	0	0
-discharge	0	0	0	0
Average points ± SD	3.33 ± 2.06	1.33 ± 1.03	0.66 ± 1.03	0

## Data Availability

Data are contained within the article.
